# Medico-legal dispute resolution: Experience of a tertiary-care hospital in Singapore

**DOI:** 10.1371/journal.pone.0276124

**Published:** 2022-10-14

**Authors:** Lee Theng Lim, Wanlin Chen, Thomas Wing Kit Lew, Jackie Mui Siok Tan, Seow Kiak Chang, Daryl Zhang Wei Lee, Thomas Swee Guan Chee

**Affiliations:** 1 Office of Clinical Governance, Tan Tock Seng Hospital, Singapore, Singapore; 2 National Healthcare Group, Singapore, Singapore; 3 Department of Anaesthesiology, Intensive Care & Pain Medicine, Tan Tock Seng Hospital, Singapore, Singapore; 4 Department of Diagnostic Radiology (Clinical), Tan Tock Seng Hospital, Singapore, Singapore; Sapienza University of Rome: Universita degli Studi di Roma La Sapienza, ITALY

## Abstract

**Introduction:**

The resource burden of healthcare disputes and medico-legal claims has been rising. A dispute resolution system operating at the hospital level could ameliorate this disturbing trend.

**Methods:**

This is a retrospective observational study on patient complaints and medico-legal cases received by the dispute resolution unit of an acute tertiary hospital from 2011 to 2015. We described the characteristics and analysed the resolution methodology and outcomes of all closed medico-legal cases.

**Results:**

Patient complaints significantly increased at a compound annual growth rate (CAGR) of 4.2% (p<0.01), while medico-legal cases and ex-gratia payments for case settlements decreased at CAGRs of 4.8% (p<0.05) and 15.9% (p = 0.19), respectively. Out of 237 closed medico-legal cases, 88.6% were resolved without legal action, of which 78.1% were closed without any ex-gratia payments or waivers. Of the 11.4% of medico-legal cases that involved legal action, 66.7% were settled without ex-gratia payments or waivers. The primary resolution modes were the Patient Relations Service (PRS)’s engagement of the complainants and facilitation of written replies. No cases were brought to court. Cases were more likely resolved without legal action when there was engagement by the PRS (p = 0.009). These cases incurred a lower median settlement value than those with legal action.

**Conclusion:**

Our hospital-based dispute resolution system which addressed patients’ core dissatisfactions and providers’ perspectives, through a process of early engagement, open disclosure, and fair negotiations, was able to promote claims resolution before legal action was taken. This early dispute resolution strategy contained costs and maintained provider-patient relationships and complements system-level mediation and arbitration to reduce medico-legal litigation.

## Introduction

There is an increasing trend of medical negligence claims with considerable financial impact to healthcare systems [1–3]. Traditionally, providers had responded with a ‘deny and defend’ stance, in the belief that this will frustrate opportunistic litigations and claims [4]. Claims which lack evidence of error are not uncommon [5]. Studdert et al, reporting in the NEJM, showed that more claims arise from medical mal-occurrence (i.e., bad outcomes) than malpractice [5]. Indeed, many medical complaints and claims were related to misguided allegations [6], poor communications, broken provider-patient relationships, and unmet expectations [7–9].

In recent times, legal jurisdictions have also established tort reforms, mediation, specialist arbitration panels, and pre-litigation ‘cooling-off’ protocols to dampen the rush to medico-legal court proceedings [10–13]. Nevertheless, these mechanisms are consequential to the breakdown of relationships and communications between patients and providers in the health systems.

Research demonstrates that a well-designed dispute resolution process emphasizes the identification of the parties’ interests and the avoidance of reliance on rights-based methods such as litigation and arbitration [14]. In some health systems, alternative dispute resolution (ADR) models have been established, which focus on less adversity and legalese in early engagement and open disclosure, and accord greater autonomy and confidentiality to resolve concerns through facilitated exchange of information and clarifications [4,10,15–19]. These in-hospital mechanisms have bridged communication and expectation-gaps in disputes, avoided litigation, reduced cost, and increased a sense of satisfaction and redress among the disputants [10,20,21].

To be effective, ADR models must also be part of a broader shift towards designing adverse-events governance and prevention at the health system level. This was widely adopted after the Leape report, which led to greater transparency, patient-centredness, and pro-active risk management systems that will detect, reduce, and prevent medical errors through iterative improvement cycles [22–24].

We describe our hospital-based dispute resolution model over a 5-year period in a large 1700-bed acute care tertiary hospital in Singapore, which has a legal jurisprudence rooted in English common law. We analyse its overall performance and the impact of resolution modalities employed in avoiding medico-legal cases.

## Materials and methods

### The dispute resolution framework and system

**Fig 1:**
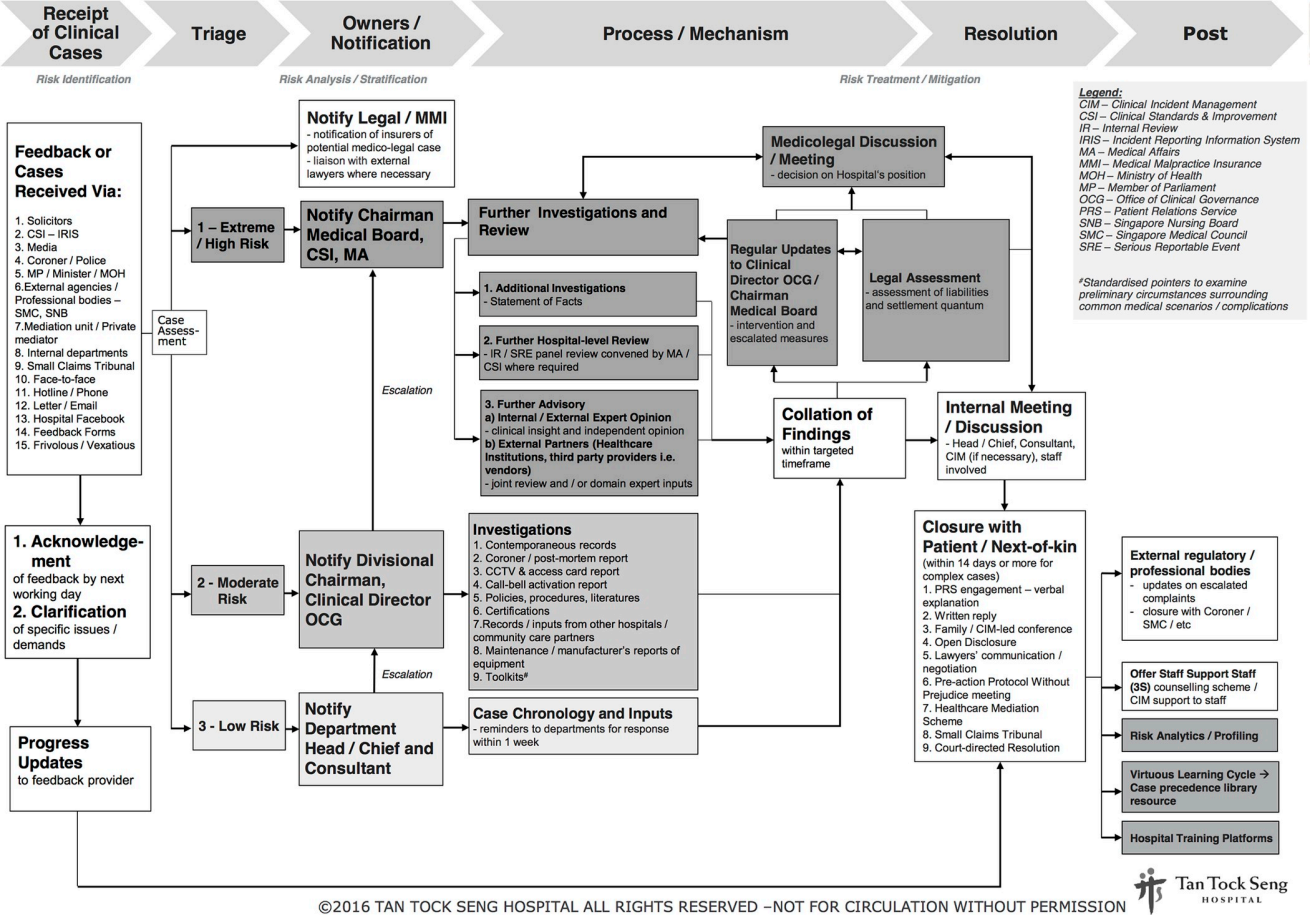
Clinical complaints and medico-legal resolution framework.

Fig 1 illustrates the framework for managing all clinical complaints, alleged and potential medical negligence, professional misconduct, and medico-legal disputes and claims. This is undertaken by a 10-person Patient Relations Service (PRS) unit, under the hospital’s Office of Clinical Governance (OCG), which reports to the chairman of the medical board (or chief medical officer) of the hospital. (The PRS unit does not handle administrative, billing or hospitality-related complaints. These are handled by a separate service-recovery unit).

Clinical incidents and complaints are received through multiple channels, including internal reporting referrals, social media platforms, external agencies and regulators. Incidents are logged and preliminarily stratified into “high-”, “moderate-” or “low-risk” categories in accordance with the common Severity Assessment Coding model [25,26], and consistent with the severity and / or reversibility of the complaint or potential grievances. These are cascaded to appropriate levels of clinician leaders, including department heads / chiefs, divisional chairpersons, clinical director of OCG, and chairman of the medical board, for rapid review and interventions. The medical malpractice insurers are concurrently notified at the outset of any potential claim for early assessment and provision of indemnity. A national Sentinel Event Root-Cause Analysis (RCA) may be undertaken concurrently by another unit in OCG for serious adverse events. The RCA is privileged and protected by law, under the Singapore Private Hospitals and Medical Clinics Regulations [27].

**Fig 2:**
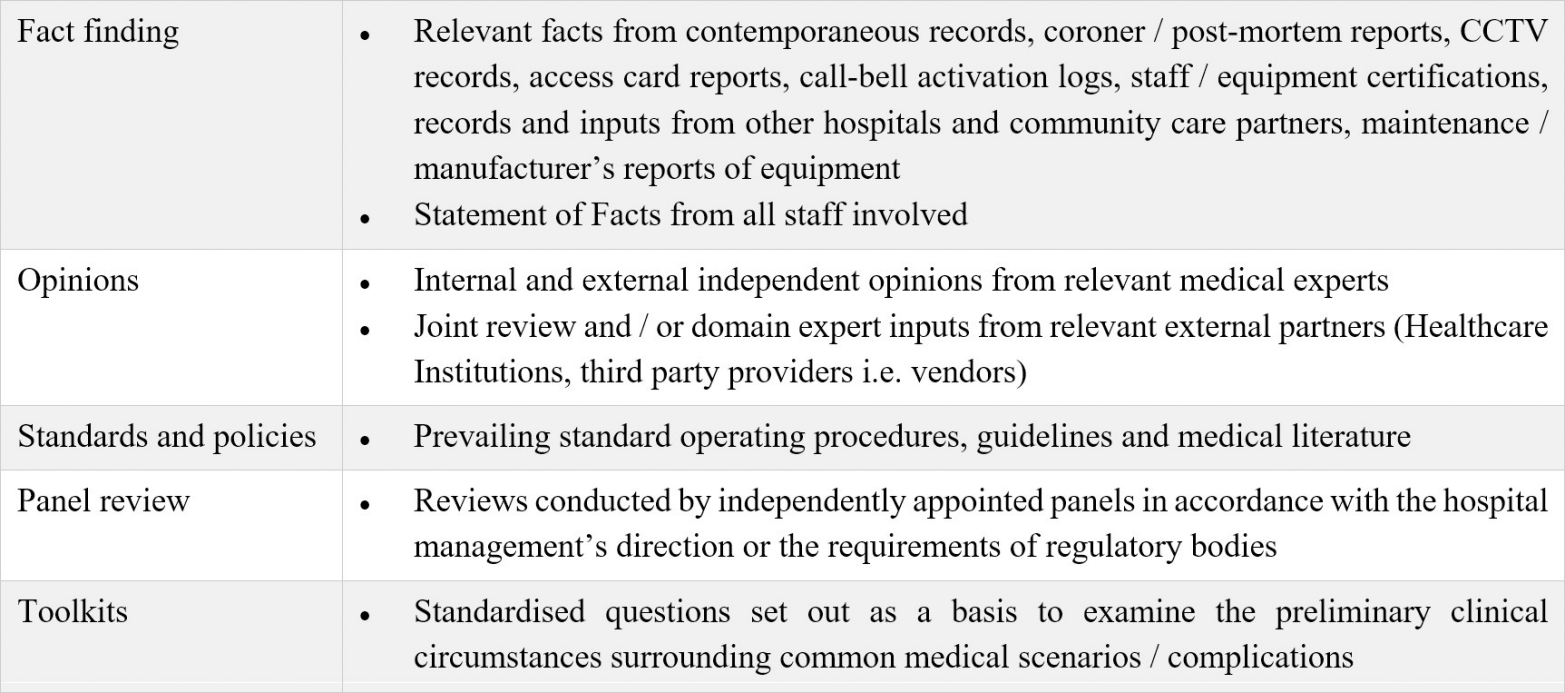
Investigation and review procedures.

Investigation and review procedures are employed systematically and as directed by leadership (Fig 2). Clinical teams managing the patients participate fully in the incident investigation and fact-finding. Detailed chronological events are painstakingly collated and resolved for ambiguities or discrepancies. Independent medical experts may be sought for neutral expert opinions to align any divergent clinical stances among providers. The relevant findings with legal advice are consolidated and reviewed by leadership for potential liability and professional accountability of the hospital. Just compensation and restitution are guided by legal precedents and contributed by the institution and the involved parties’ insurers where applicable. For unmeritorious claims, which are unsubstantiated by facts, lacking in substance, or present no rational arguments based upon the evidence in support of the claim [28], the hospital robustly defends its practices and its health professionals.

The conciliatory role played by trained PRS staff in early and active engagement of the staff, patients, and their spokespersons is a critical element in the framework. After adverse events, patients and their families experience a complex range of emotional responses, including mistrust and diatribes. PRS staff facilitate an open disclosure stance and play an intermediary role between complainants and providers. They engage the former to genuinely discern and acknowledge their perspectives, needs, and interests through empathetic listening, and a reasoned balanced approach. They address any expectation- or information-gaps, polarized views, or feelings of inequity and injustice by patients and families. A negotiated course of action, incorporating needs- and interest-based responses, targeted jargon-free explanations coalesced from the clinical teams’ inputs and hospital’s review, assurance of corrective or improvement measures, and appropriate restitution, are communicated to the complainants. Concurrently, psycho-emotional or bereavement support services by trained Medical Social Workers are proactively offered to the affected patients and family members.

### Resolution modalities

**Fig 3:**
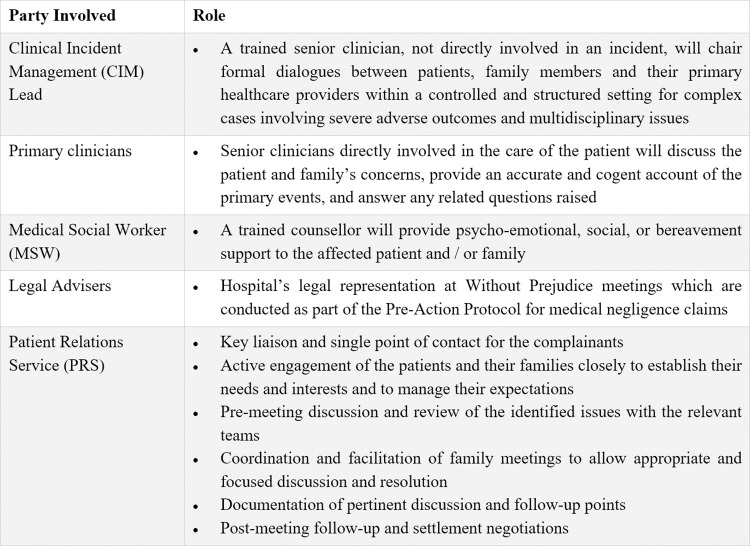
Interdisciplinary family meeting.

A PRS case manager is assigned throughout the process of resolution of all cases. We define resolution mode as the key engagement platform where the hospital’s findings, outcomes of the hospital’s investigations and apology, where applicable, were substantially communicated to the patient and their next-of-kin. A case may be resolved through the utilisation of one or more modes of resolution. They include direct bedside meetings between the clinical teams and the patients and / or their caregivers, PRS’ verbal engagement of the complainants over the phone or in person, and formal written hospital replies facilitated through the PRS unit. Face-to-face interdisciplinary-providers’ meetings with family may be chaired by trained clinical-incident management (CIM) clinicians not directly involved in the case (Fig 3). Hospital lawyers’ communications, negotiation, and pre-action without prejudice meetings may be convened under the ambit of the State Courts’ protocol for medical negligence cases [29]. Other channels of dispute resolution offered to families include facilitated mediation schemes and state-administered small claims tribunal. Some cases did not require engagement of the patient or the family members as they were flagged internally to PRS’ attention and did not materialise into formal complaints or claims, or were concluded with a State Coroner’s Inquiry. In the absence of settlement, a civil suit commenced by the patient may eventually proceed to a full trial and judgment in the court system.

Quality initiatives and system improvement measures which include clinical training and education, staff orientation and briefing, and process and policy enhancements, are undertaken to ensure prevention of errors and continuous improvement in care standards and patient safety.

### Data collection

We conducted a retrospective observational study of all patient complaints and medico-legal cases managed by the hospital between 2011 and 2015. Medico-legal cases are defined as all cases relating to both medicine and law / litigation, including (i) cases involving alleged or potential care lapses with likelihood of an anticipated claim and hence legal advice was obtained; (ii) cases where waivers and ex-gratia payments (goodwill payment made to the patients without legal obligation or admission of liability) are provided for amicable settlement; (iii) cases which have escalated to litigation. All administrative, billing or hospitality-related complaints, which are not handled by PRS, are excluded.

All medico-legal cases, which were closed by 2017, are included. They are ‘closed’ when a negotiated settlement or an amicable closure has been achieved, or no further development has occurred after 3 years from the time the primary incident is known to parties, and legal action is unlikely foreseeable or permissible under the Limitation Act. Data retrieved from the medico-legal case files which chronicled the entire case management process included all written and verbal correspondences with complainants, communications and findings from all internal and external stakeholders and third parties, minutes of meetings, relevant patient records, medical reports, statements, audio / video logs, bills, and settlement documents.

The characteristics, resolution methodology and outcomes of closed medico-legal cases were examined. Classification of the cases was performed by four case managers independently using a two-stage qualitative content analysis. In the first stage, all cases were organised by year and distributed among the case managers. For each medico-legal case, details of the patient demographics, allegation type, involved clinical discipline, mode of resolution, and case outcome, including the value of settlement, if any, were recorded. The types of allegations identified in the medico-legal cases were mainly categorised according to a taxonomy of primary incident / allegation type published in recent literature [1]. The settlement value includes any ex-gratia payment, waiver and / or token goodwill gesture made in full and final settlement of the case. At the second stage, the case managers reviewed and carried out a similar qualitative analysis of cases of a different year than the ones they examined earlier. Divergences that emerged were further discussed and harmonised through joint reviews and multiple meetings which may involve the third and fourth case manager where required to reach a consensus on the finalised classifications.

The study was performed as part of a service evaluation and audit of the hospital’s internal framework and exempted from review by the institutional group review ethics board.

### Statistical analysis

Pearson chi-square test was used to evaluate trends in the rates of patient complaints, medico-legal cases and ex-gratia payments made to settle the cases. *P*-value was calculated using the number of patient encounters (a composite of all admissions and Emergency Department attendances) as the denominator. Two-sided Pearson chi-square and Fisher’s exact tests were used to examine the associations between resolution modes and outcomes in cases closed with and without legal action. Mann Whitney U test was used to compare the difference in the value of cases settled with and without legal action. Data were analysed with SPSS version 27.

We formulated our manuscript according to the SQUIRE reporting guidelines [30]. A *p*-value of <0.05 was considered statistically significant.

## Results

From 2011 to 2015, the compound annual growth rate (CAGR) for the hospital patient encounters was 1.2%, while that for the incidence rates of patient complaints was 4.2% (p<0.01). The incidence rate of medico-legal cases showed CAGR of -4.8% (p<0.05) and the number of ex-gratia payments showed CAGR of -15.9% (p = 0.19) (Table 1).

**Table 1:** Overall trends from year 2011 to 2015.

			Year					
	2011	2012	2013	2014	2015	Total no.	CAGR	*p–*value^*^
**Patient encounters**	210,095	218,240	226,790	221,424	220,200	1,096,749	1.2%	
**Patient complaints**	2904	2886	2954	3074	3423	15241	4.2%	<0.01
**Medico-legal cases**	50	34	58	65	41	248	-4.8%	0.02
**Ex-gratia payments**	12	6	10	4	6	38	-15.9%	0.19

*Pearson chi-square test of the trend of the numbers over time.

In total, 237 out of 248 medico-legal cases were closed as of 2017. Most index patients were male (56.5%, n = 134), Chinese (79.7%, n = 189), and aged between 51 and 80 (56.5%, n = 134) (Table 2). General Surgery (21.1%, n = 50), Emergency Department (19.4%, n = 46) and Nursing Services (17.7%, n = 42) were most involved in medico-legal cases (Table3). The predominant allegation types were surgical or invasive procedures (31.2%, n = 74), treatment (16.0%, n = 38) and diagnosis (15.6%, n = 37). There were rising trends in patient falls (8.4%, n = 20) and perceived shortcomings in patient monitoring (6.8%, n = 16).

**Table 2:** Characteristics of patients (n = 237).

Patient demographics	Frequency (%)
**Age group**	
<30 (years)	22 (9.3)
31–50	38 (16.0)
51–80	134 (56.5)
>80	43 (18.2)
**Gender**	
Male	134 (56.5)
Female	103 (43.5)
**Ethnicity**	
Chinese	189 (79.7)
Malay	7 (3.0)
Indian	32 (13.5)
Eurasian	1 (0.4)
Others	8 (3.4)

**Table 3:** Characteristics of closed medico-legal cases (n = 237).

**Characteristic**	**Frequency (%)**
**Type of allegation**	
Surgical / invasive procedure	74 (31.2)
Treatment	38 (16.0)
Diagnosis	37 (15.6)
Fall	20 (8.4)
Monitoring	16 (6.8)
Medication errors	12 (5.1)
Equipment / therapeutic device	9 (3.8)
Consent	2 (0.8)
Others*	29 (12.2)
**Clinical discipline** ^**†**^	
General Surgery	50 (21.1)
Emergency Department	46 (19.4)
Nursing	42 (17.7)
Orthopaedic Surgery	25 (10.5)
General Medicine	20 (8.4)
Urology	18 (7.6)
Radiology	15 (6.3)
Ophthalmology	15 (6.3)
Anaesthesiology	15 (6.3)
Cardiology	13 (5.5)
Otorhinolaryngology	9 (3.8)
Respiratory Medicine	8 (3.4)
Neurology	7 (3.0)
Gastroenterology	7 (3.0)
Others^‡^	49 (20.7)
**Case outcomes**	
Closed without settlement	102 (43.0)
Closed with token gesture	62 (26.2)
Closed with ex-gratia and / or waiver	46 (19.4)
Legal Action–No settlement	18 (7.6)
Legal Action–Out-of-court settlement	9 (3.8)

*Allegation types which were less frequently involved in medico-legal cases, including intravenous-related complications, nosocomial infections and injuries sustained during transfers.

^†^Total percentages exceed 100% as more than one clinical discipline may be involved in a medico-legal case.

^‡^Disciplines which were less frequently involved in medico-legal cases, including Geriatric Medicine, Infectious Diseases, Radiation Oncology, Rehabilitation Medicine, Renal Medicine, Rheumatology, Allergy & Immunology, and Neurosurgery.

All closed medico-legal cases were amicably resolved. The majority (76.8%, n = 182) were closed without any payments, or with token goodwill gestures. Most cases (88.6%, n = 210) were resolved before any legal action. Of these, 78.1% (n = 164) were closed without any ex-gratia payments, waivers or settlements, while 21.9% (n = 46) were closed with formal deeds of agreement involving ex-gratia payments and waivers. Of the 11.4% (n = 27) of cases that resulted in legal escalation, 66.7% (n = 18) were closed without any settlement, while 9 cases had closure effected through out-of-court settlement agreements with ex-gratia payments and waivers.

Associations between resolution modes and medico-legal cases closed with and without legal action were also examined (Table 4). The two primary modes of case resolution were the PRS unit’s engagement of the complainants to provide verbal explanation of the findings of the hospital’s investigations (35.0%, n = 83), and facilitation of formal letters detailing the hospital’s findings and position (54.9%, n = 130). Other resolution mechanisms include clinicians-led family meetings (22.8%, n = 54) and direct team engagement (15.6%, n = 37), where the managing team addressed complaints at patients’ bedside or at outpatient setting. Less frequently, family meetings were facilitated by independent CIM-trained clinicians (9.7%, n = 23). There was a small number of medico-legal cases (10.5%, n = 25) that did not require direct engagement of the patient or the family members.

**Table 4:** Association between resolution modes and medico-legal cases closed with and without legal action.

Resolution modes	Cases closed without legal action n = 210	Cases closed with legal action n = 27	*p—*value
**Bedside / direct team engagement, n = 37** No Yes	174 (82.9) 36 (17.1)	26 (96.3) 1 (3.7)	0.090^†^
**PRS engagement, n = 83** No Yes^‡^	130 (61.9) 80 (38.1)	24 (88.9) 3 (11.1)	0.009*
**Written reply, n = 130** No Yes	95 (45.2) 115 (54.8)	12 (44.4) 15 (55.6)	>0.950*
**Family meeting, n = 54** No Yes	162 (77.1) 48 (22.9)	21 (77.8) 6 (22.2)	>0.950*
**CIM-led family meeting, n = 23** No Yes	190 (90.5) 20 (9.5)	24 (88.9) 3 (11.1)	>0.950^†^
**Lawyers’ communication / negotiation, n = 21** No Yes	210 (100.0) 0 (0.0)	6 (22.2) 21 (77.8)	<0.001^†^
**Without Prejudice meeting, n = 9** No Yes	210 (100.0) 0 (0.0)	18 (66.7) 9 (33.3)	>0.950^†^
**Mediation / Small claims tribunal, n = 3** No Yes	207 (98.6) 3 (1.4)	27 (100.0) 0 (0.0)	>0.950^†^
**No engagement required, n = 25** No Yes	185 (88.1) 25 (11.9)	27 (100.0) 0 (0.0)	0.088^†^
**Court trial / judgement, n = 0** No Yes	27 (100.0) 0 (0.0)	210 (100.0) 0 (0.0)	-

*Pearson chi-square test

^†^Fisher’s exact test.

^‡^PRS staff engagement of the complainants over the phone or in person.

In cases where legal action had commenced, the hospital’s lawyers typically facilitated the subsequent processes, including letters of replies and negotiation (77.8%, n = 21). It is noteworthy that written replies issued by the hospital (55.6%, n = 15) were predominant in these cases. A few cases (33.3%, n = 9) involved without-prejudice meetings conducted by the solicitors representing the complainants and the hospital under the State Courts’ protocol for medical negligence claims. No case proceeded to a full trial for judgment in the law courts. One dispute was brought to the small claims tribunal but was discontinued because the nature of the complaint was outside its jurisdiction. Two complaints were referred to the government health-regulator’s Healthcare Mediation Unit and were resolved amicably.

A significant association was found between PRS engagement and all closed medico-legal cases (p = 0.009). With the utilisation of PRS engagement, medico-legal cases were more likely to be resolved without legal action [n (%) with legal action versus without legal action: 3 (11.1%) vs. 80 (38.1%)].

Cases involving legal action had a significantly higher median settlement amount (SGD$14,500.00) compared to cases resolved without legal action (SGD$2,110.08) (p = 0.001).

## Discussion

ADR models can be broadly categorised as in-built within health systems, as described in this study, or external processes implemented by legal jurisdictions, health regulators, and insurers. Boothman, Lin, Farber and others have shown that in-built systems in healthcare institutions to manage complaints and undertake direct negotiations between healthcare providers and complainants, were effective in resolving such disputes and preventing litigation. Importantly, they were the preferred and most efficient means of resolving disputes, as opposed to ADR approaches involving external agencies and litigation [4,16,19,31].

The characteristics of the medico-legal cases in our study were similar to previous studies in UK, Australia and the United States, for incident-types, and high-risk medical specialties involved [1,2,32–35]. Against a tide of rising numbers of complaints (CAGR4.2%), the rate of medico-legal cases declined significantly (CAGR -4.8%) and ex-gratia payments showed a downward trend (p = 0.19).

Our results are similar to the experience of the University of Michigan Health System (UMHS)’s in-house ADR program, which saw its number of settlements decrease, and its overall claims drop by 55% from 1999 to 2006, despite an increase in case management and clinical activities over the same period [4,36].

Most cases (88.6%) in our study were resolved without legal action. Of the remaining 11.4% which resulted in legal action, none were brought to court (Table 4). This compares favourably with an earlier study by Farber and White reporting on an informal dispute resolution model adopted by a single large general hospital, which managed to close 45.3% of its cases without legal action while 3.1% of the cases resulted in a full trial and judgement [16].

In our study, the median settlement amounts of the medico-legal cases that were resolved without legal action were significantly lower than those with legal action (p<0.001). This represents significant cost-savings including the avoidance of legal defence expenses, and resonates with the study by Farber, which reported that the settlement amounts of cases resolved before legal action were significantly lower than that of cases resolved after a lawsuit was filed [16]. In our system, the quantum of the settlement is negotiated fairly at the local hospital level, and based on legal advice and established principles for compensatory damages such as causation, loss and mitigation. In other jurisdictions, decisions on financial pay-outs are made by external agencies, such as NHS Resolution in the UK (formerly known as the NHS Litigation Authority), which oversees and deals with clinical negligence claims on behalf of all NHS trusts and organisations [15].

COPIC Insurance Company, a Colorado-based medical liability carrier, had also introduced in 2000 an early intervention program for “recognize, respond, and resolve” (“3Rs”) which emphasized disclosure, transparency, apology, and patient benefits. By 2008, they had reported 3,000 events, of which two-thirds were closed with no payment to the patient. Malpractice claims against COPIC physicians reportedly dropped by 50% and settlement costs dropped 23% [37]. Such external ADR models by malpractice insurers do not however have direct mechanisms to link learning from the analysis and outcome of such events to prevention and improvement programs compared to ADR models within health systems. In our study, there was a very low percentage of external ADR mechanisms utility (1.3%; n = 3), suggesting that the in-built dispute resolution mechanisms for early intervention and engagement was effective and well accepted by patients for most medico-legal cases.

In our study, a significant association was found between PRS’ engagement of the complainants over the phone or in person to provide substantive explanations of the hospital’s findings, and all closed medico-legal cases (p = 0.009). PRS’ role encompasses a deep understanding of the hospital’s operating systems and a trusted relationship with clinicians, while maintaining strong patient advocacy. In this resolution modality, PRS seeks to provide needs- and interest-based responses and targeted explanations to sincerely address any expectation- or information-gaps, polarized views, or feelings of inequity and injustice by patients and families. We believe this conciliatory and intermediary role played by PRS through early and active engagement of staff, patients and spokespersons is an integral structure of the model, and was able to promote case resolution before legal action was taken.

Formal and comprehensive written communication by the hospital senior clinician leaders was the most common resolution modality, used in 54.9% of cases. This modality reflects the organisation’s belief that by providing a comprehensive and accurate account of the incident, patients or their next-of-kin would be able to seek expert opinions or legal advice based on its content, and the hospital’s fair and just medicolegal position would be transparent, leading to an early resolution. In our series, 76.8% of all cases were closed without any payments, or with token goodwill gestures such as modest fee waivers and reimbursement.

There are several limitations in this report. Manpower resources and legal costs were not included in this study but are nevertheless a key measure of cost effectiveness in the management of medico-legal cases. Legal advice in support of ADR models is generally less costly than the defense of litigation in court. However, time and resources are needed to resolve complex cases [38]. The comparison of the median settlement amounts merely provides a general insight into the values of the pay-out made to the patients or families when a case is negotiated directly by the hospital or through legal action. Lastly, medico-legal cases often entail a complex interplay of patient, healthcare professional and societal factors, and the findings of our study should be interpreted with care when applied to other settings.

In conclusion, the lack of an explanation about an injury can precipitate a malpractice claim as patients instinctively turn to litigation for answers; full apologies, on the other hand, promote settlement [39,40]. In 2014, the Singapore Chief Justice similarly opined that the tort of negligence and the adversarial nature of the litigation system do not provide a holistic solution for medical disputes. He emphasized that patients often initiate legal proceedings because of emotional reactions or unrealised expectations, and that they may not necessarily be seeking financial gain, but for an explanation, or a sincere gesture of apology or empathy [20]. Our experience adds to the literature that health systems can effectively remediate these deficiencies. Based on our evaluation of our dispute resolution system, the overall outcomes are positive and there are advantages in having an organic hospital unit to coordinate and resolve claims and medico-legal disputes. Such an effective in-built dispute resolution system can potentially avert litigation, contain costs, and bring about restoration, renewed trust and continuous organisational learning.

## Supporting information

S1 Raw data (XLSX)
